# A genome-wide analysis of MADS-box genes in peach [*Prunus persica* (L.) Batsch]

**DOI:** 10.1186/s12870-015-0436-2

**Published:** 2015-02-07

**Authors:** Christina E Wells, Elisa Vendramin, Sergio Jimenez Tarodo, Ignazio Verde, Douglas G Bielenberg

**Affiliations:** Department of Biological Sciences, Clemson University, Long Hall, 29634 Clemson, SC USA; Consiglio per la Ricerca in Agricoltura e l’analisi dell’economia agraria, Centro di Ricerca per la Frutticoltura (CRA-FRU), Rome, Italy; School of Agriculture, Forestry and Life Sciences, Clemson University, Poole Agricultural Center, 29634 Clemson, SC USA

**Keywords:** MADS-box gene, MIKC gene, Dormancy, Peach, *Prunus persica*, SVP, FLC, AGL24

## Abstract

**Background:**

MADS-box genes encode a family of eukaryotic transcription factors distinguished by the presence of a highly-conserved ~58 amino acid DNA-binding and dimerization domain (the MADS-box). The central role played by MADS-box genes in peach endodormancy regulation led us to examine this large gene family in more detail. We identified the locations and sequences of 79 MADS-box genes in peach, separated them into established subfamilies, and broadly surveyed their tissue-specific and dormancy-induced expression patterns using next-generation sequencing. We then focused on the dormancy-related *SVP/AGL24* and *FLC* subfamilies, comparing their numbers and phylogenetic relationships with those of other sequenced woody perennial genomes.

**Results:**

We identified 79 MADS-box genes distributed across all eight peach chromosomes and frequently located in clusters of two or more genes. They encode proteins with a mean length of 248 ± 72 amino acids and include representatives from most of the thirteen Type II (MIKC) subfamilies, as well as members of the Type I Mα, Mβ, and Mγ subfamilies. Most Type I genes were present in species-specific monophyletic lineages, and their expression in the peach sporophyte was low or absent. Most Type II genes had *Arabidopsis* orthologs and were expressed at much higher levels throughout vegetative and fruit tissues. During short-day-induced growth cessation, seven Type II genes from the *SVP/AGL24*, *AGL17,* and *SEP* subfamilies showed significant changes in expression. Phylogenetic analyses indicated that multiple, independent expansions have taken place within the *SVP/AGL24* and *FLC* lineages in woody perennial species.

**Conclusions:**

Most Type I genes appear to have arisen through tandem duplications after the divergence of the *Arabidopsis* and peach lineages, whereas Type II genes appear to have increased following whole genome duplication events. An exception to the latter rule occurs in the *FLC* and *SVP/AGL24* Type II subfamilies, in which species-specific tandem duplicates have been retained in a number of perennial species. These subfamilies comprise part of a genetic toolkit that regulates endodormancy transitions, but phylogenetic and expression data suggest that individual orthologs may not function identically across all species.

**Electronic supplementary material:**

The online version of this article (doi:10.1186/s12870-015-0436-2) contains supplementary material, which is available to authorized users.

## Background

Seasonal dormancy is an endogenous repression of meristematic growth exhibited by many perennial plants during the cold winter months. Endodormancy entrance and release are triggered by day length and/or temperature cues using a regulatory network that shares key features with the vernalization and photoperiodic flowering time pathways of *Arabidopsis* [1]. Nonetheless, precise mechanisms of endodormancy regulation in woody plants have not been characterized.

The peach *evergrowing* (*evg*) mutant has lost six tandem-duplicated *dormancy-associated MADS-box* (*DAM*) genes and does not form terminal buds or enter endodormancy under short day conditions [2]. The *DAM* genes are most closely related to *Arabidopsis SVP* and *AGL24*, both of which are involved in vernalization and flowering time regulation [1]. In peach, *DAM* gene expression tracks seasonal light and temperature cycles, and we have hypothesized that *DAM* genes integrate environmental cues to regulate the transition into and out of endodormancy [3]. Down-regulation of *DAM* homologs is also correlated with endodormancy release in Japanese apricot (*Prunus mume*) [4], Japanese pear (*Pyrus pyrifolia*) [5] and raspberry (*Rubus idaeus*) [6]. *FLC*, another MADS-box gene, plays a central role in *Arabidopsis* vernalization but has not been identified in dormancy-related gene sets from grape, Norway spruce, or peach [7-10].

The central role played by MADS-box genes in peach dormancy regulation has led us to examine this large gene family in more detail. MADS-box genes encode a family of eukaryotic transcription factors distinguished by the presence of a highly-conserved ~58 amino acid DNA-binding and dimerization domain at the N-terminal (the MADS-box) [11]. In plants, MADS-box genes are best known as master regulators of flowering time and floral organ development, although they also function in the development of leaves, roots, fruit, seeds and gametophytes [12,13]. Members of the MADS-box gene family are found throughout higher eukaryotes and are divided into two classes, Type I and Type II, which arose from a single gene duplication before the divergence of plants and animals [14]. Type I genes are characterized by the presence of the MADS-box and by a simple intron-exon structure, while Type II genes possess additional conserved domains and a more complex gene structure [15,16].

In plants, Type II genes are termed MIKC (MADS Intervening Keratin-like C-terminal) genes in reference to the four recognized domains of their protein products. In addition to the MADS-box, MIKC proteins possess an intervening I domain (~30 aa) that contributes to dimerization specificity, a highly-conserved keratin-like K domain (~70 aa) that facilitates dimerization, and a variable C-terminal domain that plays a role in transcriptional activation and the formation of multimeric complexes [16]. MIKC genes are further divided into MIKC^c^ and MIKC* classes, with the latter exhibiting an ancestral duplication within the K domain [17].

MIKC^c^ genes are the best-studied plant MADS-box genes and have been divided into at least 13 subfamilies based on sequence similarity [18]. Several subfamilies form the basis for the ABCDE model of floral organogenesis, in which specific combinations of genes from the *AP1, AP3/PI, AG, FUL* and *SEP* subfamilies give rise to sepals, petals, stamens, carpels and ovules in *Arabidopsis thaliana* [19]. A subset of MIKC^c^ genes from the *FLC*, *SOC1* and *SVP/AGL24* subfamilies control vernalization and flowering time in response to seasonal light and temperature cues in annual plants [20,21]. Genes from the *FLC* and *SVP/AGL24* subfamilies also appear to regulate endodormancy transitions in perennial plants, using pathways that share significant features with those of vernalization [1,4,22].

In contrast to MIKC^c^ genes, the functions of Type I and MIKC* genes are poorly understood. Recent work suggests that Type I genes are chiefly expressed in the female gametophyte and the developing seed of *Arabidopsis* [23]. Expression levels are often quite low, and there is evidence for considerable functional redundancy. MIKC* genes appear to function primarily in the *Arabidopsis* male gametophyte, where they control the expression of genes required for pollen maturity [24].

Here we present a genome-wide analysis of Type I and II MADS-box genes in peach, made possible by the availability of the peach genome sequence (Peach v1.0; [25]). We report the locations and sequences of Type I and II MADS-box genes in peach, separate them into established subfamilies, and broadly survey their tissue expression patterns. We then focus on the *SVP/AGL24* and *FLC* subfamilies, comparing their numbers and phylogenetic relationships with those of other perennial species and quantifying their expression during the transition to endodormancy in peach. In particular, we test the hypotheses that (1) a similar expansion within the *SVP/AGL24* subfamily has occurred in multiple perennial plant species and (2) genes from the *SVP/AGL24* and *FLC* subfamilies are differentially expressed during the short-day dormancy transition in peach.

## Methods

### Sequence collection

Peach genome scaffolds, predicted peptides and ESTs were obtained from the Genome Database for Rosaceae (http://www.rosaceae.org/species/prunus_persica/genome_v1.0, [25]). MADS-box protein sequences from *Arabidopsis thaliana*, *Vitis vinifera, Populus trichocarpa, Zea mays,Sorghum bicolor* and *Oryza sativa* were retrieved from Phytozome v9.1 (http://www.phytozome.net/) and named according to the conventions of Parenicova *et al.* 2003 [26], Diaz-Riquelme *et al*. 2009 [18], Leseberg *et al.* 2006 [27], Zhao *et al.* 2011 [28], and Arora *et al.* 2007 [29], respectively. An exception occurred with the *FLC* genes from *P. trichocarpa*, which were incompletely annotated in the *Populus* v3.0 genome build. These sequences were curated manually and named according to the transcript ID containing their MADS box. Our revised *Populus FLC* protein sequences are given in Additional file 1.

### Identification and annotation of peach MADS-box genes

The HMMER-3.0 software package [30] was used to build profile hidden Markov models from full Pfam alignment files for the MADS-box (SRF-TF PF00319) and K-box domains (K-box PF01486). Resulting models were used to search the database of predicted peach peptides and identify potential MADS-box proteins (E-value threshold 1 × e^−10^, with manual inspection of sequences close to the threshold). The full peach genomic scaffolds were also queried with nucleic acid sequences from representative *Arabidopsis* and *Vitis* MADS-box genes using NCBI BLAST tools [31] to identify putative MADS-box genes not present in the predicted protein set.

A 15 kb region around each peach MADS-box was extracted, and the full gene structure was predicted using the FgenesH (Softberry, Inc., Mount Kisco, NY), Augustus [32] and SNAP [33] gene prediction programs within the DNA Subway annotation pipeline (http://dnasubway.iplantcollaborative.org/). Predicted models were refined by manual inspection and comparison with homologous *Arabidopsis* sequences and peach ESTs. Positions of MADS-box genes on peach genome scaffolds were visualized with MapChart software [34] and are provided as a gff3 file in Additional file 2.

### Phylogenetic analyses

An initial phylogenetic analysis was performed to separate the peach MADS-box genes into Type I and Type II lineages. Fifty-eight amino acids from the MADS-box domain of each *Arabidopsis* and peach gene were aligned with Clustal W [35] and used to create a maximum likelihood phylogenetic tree in PhyML 3.0 [36]. Positions of MADS-box genes on the resulting tree classified them unambiguously as Type I or II, and these assignments were verified by confirming the presence of a K-box in the MIKC genes only.

Protein sequences of MIKC genes from peach and *Arabidopsis* were aligned with MAFFT v7 [37], and a phylogenetic analysis was performed with MrBayes v3.2 using the Jones amino acid substitution model [38]. Two independent runs with four Markov Chain Monte Carlo chains were run for 10 million generations and sampled every 1000 generations to achieve convergence (standard deviation of split frequencies < 0.02). After dropping the first 25% of the sampled trees as burn-in, results were visualized as a consensus tree with posterior probabilities indicated at each node. Trees were constructed in the same manner to partition Type I genes among Mα, Mβ, and Mγ clades and to analyze the relationships among genes from the *FLC* and *SVP/AGL24* subfamilies across multiple species.

### Tissue-specific expression analyses

75 base-pair paired-end Illumina RNAseq reads (llumina Inc., San Diego, CA) from root, expanded leaf, young apical leaf, fruit, pollen and cotyledon + embryo tissues were obtained as described in Verde *et al.* 2013 [25] and are available for download from the NCBI Sequence Read Archive (SRA053230). Reads were quality-trimmed using the default settings of ConDeTri [39] prior to read mapping and transcript quantification with the Cufflinks pipeline (Bowtie 1.0.0, TopHat 2.0.9, Cufflinks 2.1.0) and the peach v1.0 reference genome [25,40]. Estimated depth of transcriptome coverage was high but differed among the read sets. After filtering and trimming, the root, expanded leaf, young leaf, fruit, pollen and cotyledon + embryo read sets provided approximately 108X, 100X, 171X, 102X, 135X, and 67X coverage of the peach transcriptome, respectively. Reads from each tissue were mapped and quantified separately, using a gff3 file of peach MADS-box gene models as a reference and without assembly of additional transcripts (−G option in Cufflinks). Resulting expression values (FPKM, i.e. fragments per kilobase of exon model per million mapped fragments) were log-transformed and used in an average linkage clustering analysis with Cluster 2.11 and TreeView 1.6 in order to visualize tissue-specific gene expression patterns [41]. All expression data are provided in Additional file 3.

### Short-day expression analyses

Rooted peach cuttings were grown in a greenhouse for two months at 25°C under long days (LD, 16 h light/8 h dark). Cuttings were derived from wild type individuals in the F_2_ population described in Jimenez *et al.* 2010 [9]. Plants were transferred to a growth room for two weeks of acclimation under LD, then shifted to SD conditions (8 h light/16 h dark) for two weeks. In the growth room, 250–300 μmol m^−2^ s^−1^ of light was provided at canopy height by AgroSun® Gold 1000 W sodium/halide lamps (Agrosun Inc, New York, NY, USA). Temperatures averaged 22.5°C (light) to 18.7°C (dark), and relative humidity ranged between 48% and 55%. Plants were watered every two days as needed.

At 0, 1, and 2 weeks after the transfer to SD, apical tips (youngest leaves and shoot apical meristems) from eight replicate plants per week were harvested and pooled for RNA extraction [42]. Following quantification and quality assessment on the Agilent 2100 Bioanalyzer (Agilent Technologies, Santa Clara CA), 10 μg of ethanol-precipitated total RNA from each pooled sample was shipped to the Iowa State University DNA Facility for library preparation and 75 bp single-end sequencing on the Illumina Genome Analyzer II platform. Resulting sequence data were quality-filtered and trimmed as above prior to transcript assembly and quantification with the Cufflinks pipeline and average linkage clustering with Cluster and TreeView. Genes whose expression levels changed significantly through time were identified using the Audic and Claverie statistic implemented in IDEG6 with *P* <0.05 and a Bonferroni correction for multiple comparisons [43,44]. All expression data are provided in Additional file 3, and raw reads are available at the NCBI Sequence Read Archive (SRP046357).

## Results

### MADS-box genes in peach

We used profile hidden Markov models to identify the positions and sequences of 79 MADS-box genes in the peach genome: 40 Type I and 39 Type II. Thirteen of these genes have been described previously, and two additional genes match peach ESTs available at NCBI (Additional file 4). They encode predicted proteins with a mean length of 248 ± 72 amino acids and include representatives from most Type II (MIKC) subfamilies, as well as members of the Type I Mα, Mβ, and Mγ subfamilies. Also identified were four probable pseudogenes with premature stop codons within the first two exons. These genes (*PpeMADS02, PpeMADS05*, *PpeMADS68*, and *PpeMADS72*) were dropped from further analysis. The majority of Type I genes had a single exon, while Type II genes had between 7 and 9 exons.

The number of MADS-box genes in peach is lower than that of *Arabidopsis* (108) and poplar (101) and similar to that of sorghum (76), rice (65) and maize (75; Table 1). The larger number of MADS-box genes in *Arabidopsis* is due primarily to an expansion within the Type I Mβ clade (21, compared with 2–12 in other species), while poplar has a larger number of MIKC^c^ genes (51) than other species (32–39).

**Table 1:** **Numbers of MADS-box genes in seven sequenced plant genomes** [18,26-29]

		***Prunus persica***	***Arabidopsis thaliana***	***Populus trichocarpa***	***Vitis vinifera***	***Oryza sativa***	***Sorghum bicolor***	***Zea mays***
**Type I**		40	62	41	--	32	30	32
	**M** **α**	21	25	23	--	13	26	27
	**Mβ**	7	21	12	--	9	2	3
	**Mγ**	12	16	6	--	10	2	2
**Type II**		39	46	60	--	44	35	43
	MIKC*	4	7	9	--	6	2	4
	**MIKC** ^**c**^	35	39	51	32	38	33	39
**Grand total**		79	108	101	--	76	65	75

### Chromosome positions

**Figure 1:**
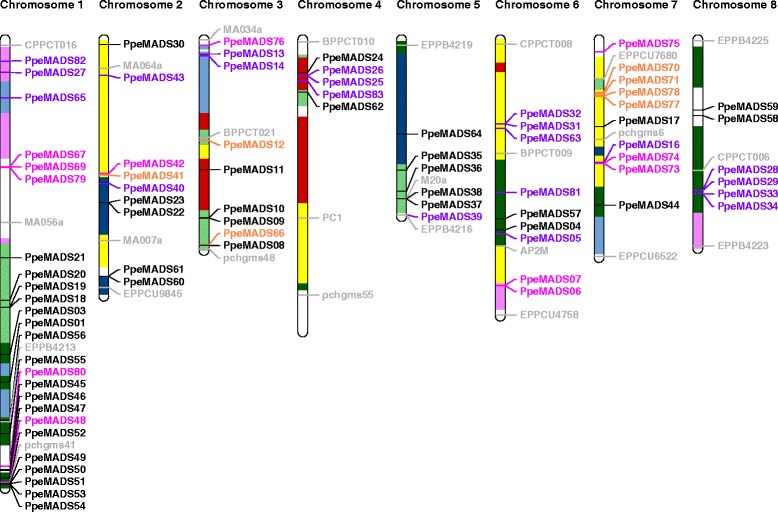
**Chromosomal locations of MADS-box genes in peach.** MIKC genes are shown in black, Mα genes in purple, Mβ genes in orange, and Mγ genes in fuchsia. Selected molecular markers are shown in gray. Seven major syntenic regions of the peach genome are indicated by colored segments on chromosome bars [25].

MADS-box genes are distributed across all eight chromosomes of peach (Figure 1). Sixty percent of the peach MADS-box genes are clustered, i.e. present in groups of two or more genes separated by fewer than 200 kb [45]. The extent of clustering is particularly high in the Type I Mβ and Mγ subfamilies, 87.5% and 84.6% of whose genes are clustered. Clusters generally consist of close paralogs, but this is not always the case. *PpeMADS66* (Mβ) and *PpeMADS08* (MIKC^c^*FLC*-like) are located within 59 kb of one another on chromosome 3, while *PpeMADS16* (Mα) is located within 86 kb of two tandem duplicated Mγs (*PpeMADS73* and *74*) on chromosome 7.

Several closely-adjacent pairs of distantly-related MADS-box genes are found multiple times in syntenic regions of the peach genome. There are three occurrences of a *SEP*-like gene located within 4 to 11 kb of a *AP1/FUL-*like gene within syntenic regions: *PpeMADS18* and *PpeMADS19* on chromosome 1, *PpeMADS09* and *PpeMADS10* on chromosome 3, and *PpeMADS37* and *PpeMADS38* on chromosome 5. Likewise, a *SOC1* and an *AGL6* homolog (*PpeMADS22* and *PpeMADS23*, *PpeMADS60* and *PpeMADS61*) are closely adjacent to one another on opposite strands at two positions on duplicated portions of chromosome 2. Such patterns have been reported previously [46] and suggest an ancient tandem duplication, followed by retention of the resulting paralogs and later duplication of the gene pair by polyploidization.

### MADS-box protein phylogenies

**Figure 2:**
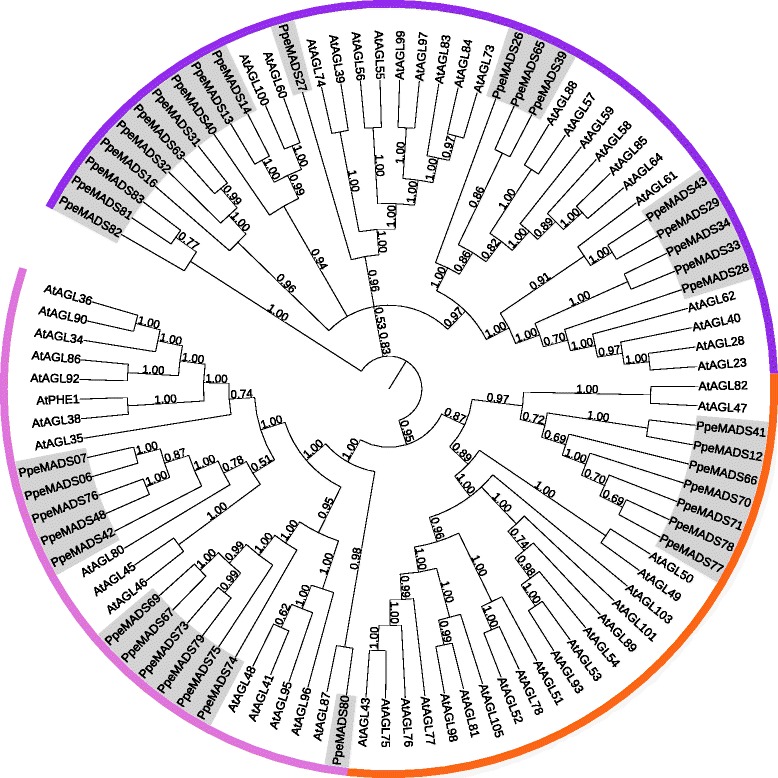
**Unrooted Bayesian consensus tree of Type I MADS-box proteins from peach and** ***Arabidopsis*** **.** Bayesian posterior probabilities for all clades are given at their respective nodes. Mα genes are shown in purple, Mβ genes in orange, and Mγ genes in pink.

**Figure 3:**
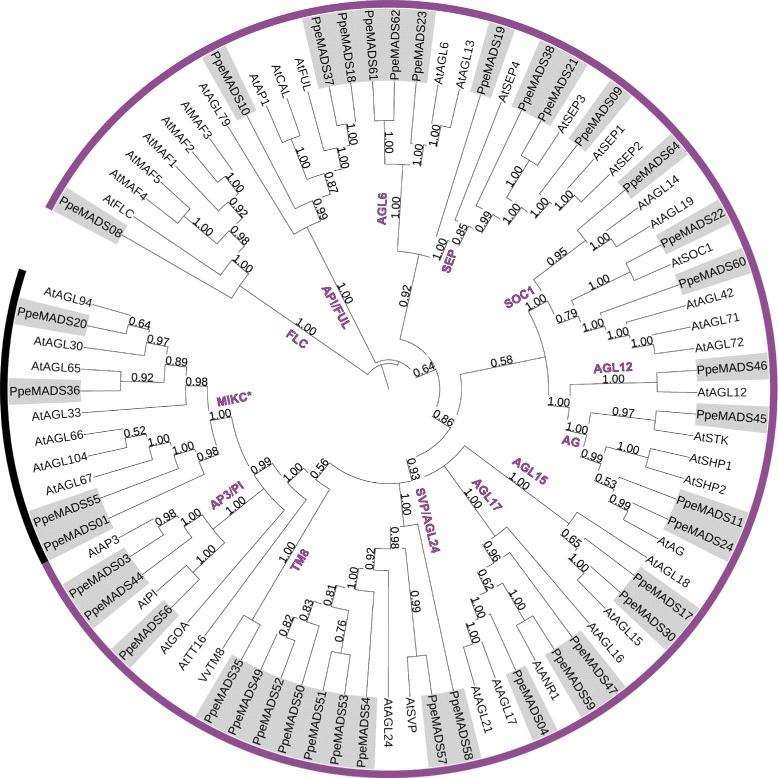
**Unrooted Bayesian consensus tree of Type II MADS-box proteins from peach and** ***Arabidopsis*** **.** Bayesian posterior probabilities for all clades are given at their respective nodes. Established Type II subfamilies are indicated in purple text, MIKC* genes are shown in black, and MIKC^c^ genes are shown in purple. MIKC^c^ subfamilies are named after [18].

Unrooted phylogenetic trees were constructed from full length protein sequences of Type I and Type II MADs-box genes of *Arabidopsis* and peach (Figures 2 and 3). Type I genes from both species grouped into the previously-identified Mα, Mβ and Mγ subfamilies with moderate support. While most Type I genes were present in species-specific monophyletic lineages, a small number of *Arabidopsis* Type I genes did have close peach orthologs. For example, the central cell-expressed Mα *AGL61* (*DIA*) has two peach orthologs (*PpeMADS29* and *PpeMADS43*), while its Mγ interaction partner *AGL80* has five peach orthologs (*PpeMADS06, PpeMADS07, PpeMADS42, PpeMADS48* and *PpeMADS76*).

Type II genes grouped into MIKC* and MIKC^c^ clades, the latter containing members from 12 established subfamilies (Figure 3; [18]). The majority of Type II subfamilies contained similar numbers of genes in *Arabidopsis* and peach. Exceptions occurred in the two subfamilies that play a pivotal role in *Arabidopsis* vernalization and flowering time: *SVP/AGL24* and *FLC*. In *Arabidopsis*, the *SVP/AGL24* subfamily contains only the two eponymous genes. In peach, the family is expanded to eight genes: the six *DAM* genes (*AGL24* orthologs), *PpeMADS57* (an *SVP* ortholog), and *PpeMADS58*, which has no *Arabidopsis* ortholog. Conversely, the *FLC* subfamily contains six members in *Arabidopsis* (*FLC* and *MAF1-5*) but only a single member in peach (*PpeMADS08*).

**Figure 4:**
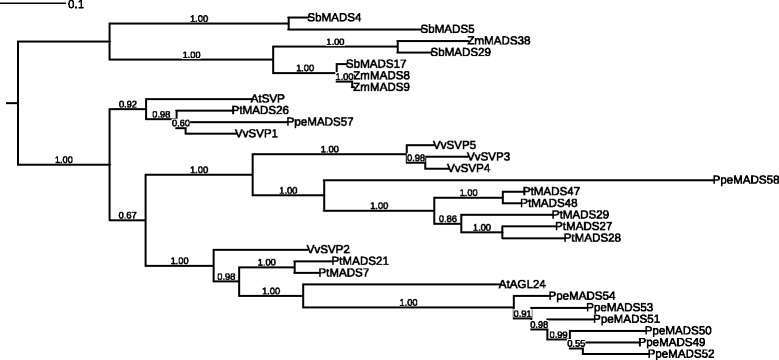
**Unrooted Bayesian consensus tree of MADS-box proteins from the SVP/AGL24 subfamily in peach,** ***Arabidopsis*** **, grapevine, poplar, maize, sorghum, and rice.** Bayesian posterior probabilities for all clades are given at their respective nodes.

To further investigate gene numbers and relationships within the *SVP/AGL24* and *FLC* subfamilies, we created phylogenetic trees of *SVP/AGL24* and *FLC* proteins from seven plant species with sequenced genomes and fully-catalogued MIKC^c^ genes: *Arabidopsis* [26]*,* peach, poplar [27], grapevine [18], maize [28], sorghum [28] and rice [29]. It is clear that multiple independent expansions have occurred within the *SVP/AGL24* subfamily over the course of eudicot evolution (Figure 4). While the peach *DAM* gene expansion (*PpeMADS49-54*) occurred within the *AGL24* lineage, expansions in poplar and grapevine have taken place in a separate lineage that contains *PpeMADS58* and no *Arabidopsis* members. Monocot *SVP/AGL24* homologs form a completely separate lineage with 2–3 members per species.

**Figure 5:**
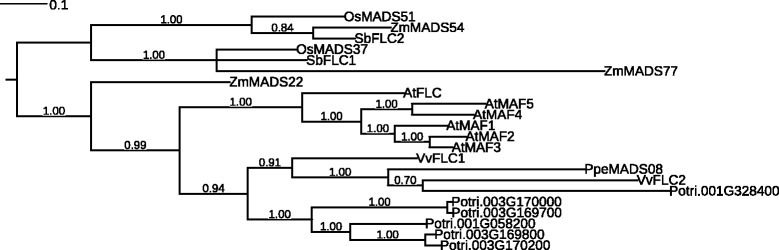
**Unrooted Bayesian consensus tree of MADS-box proteins from the FLC subfamily in peach,** ***Arabidopsis*** **, grapevine, poplar, maize, sorghum, and rice.** Bayesian posterior probabilities for all clades are given at their respective nodes.

The *FLC* subfamily is expanded in *Arabidopsis* by the presence of the 5 *MAF* genes, which have no orthologs in any other species examined (Figure 5). The *FLC* subfamily contains two to three members in monocots, one in peach, two in grapevine and six in poplar. The single peach *FLC*-like gene (*PpeMADS08*) belongs to a lineage separate from that of *Arabidopsis FLC* and the *MAFs*, while five *FLC*-like genes from poplar form a species-specific clade. Expansions of the *FLC* gene family in *Arabidopsis* and poplar are clearly the result of separate evolutionary events.

Peach contains a single member (*PpeMADS35*) of the *TM8* subfamily, a group of floral development genes present in many eudicots but lost in *Arabidopsis* [47]. Like many other eudicots, peach also has third member of the *AP3/PI* subfamily. Peach does not appear to contain members of the *Bsister* subfamily, represented by *GOA* and *TT16* in *Arabidopsis*.

### Tissue-specific gene expression

**Figure 6:**
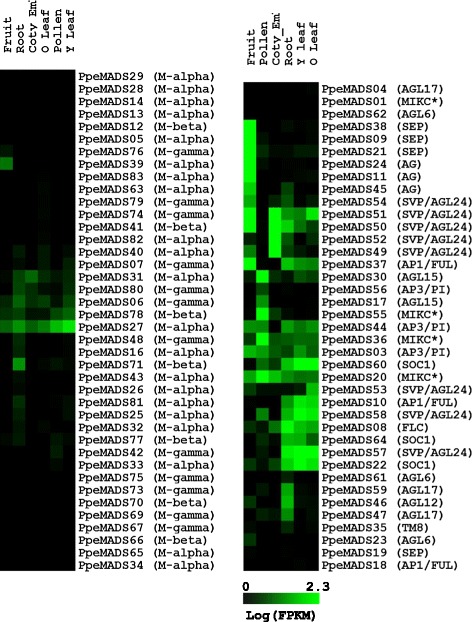
**Expression profiles of Type I (left) and Type II (right) MADS-box genes from six peach tissues: root, expanded leaf (O Leaf), young leaf (Y leaf), fruit, pollen and cotyledon + embryo (Coty_embryo) tissue.** FPKM expression values were log-transformed, and genes were grouped by average linkage clustering (see Methods).

RNA-seq data were used to quantify the expression MADS-box genes in six peach tissues (Figure 6). Expression of Type I genes was generally low or absent. Among 40 Type I genes, 14 showed no expression and only six were expressed at levels higher than 2 FPKM in any tissue. A notable exception to this pattern was *PpeMADS27*, an Mα gene detected at moderate levels in all tissues (2.4-19.3 FPKM), particularly young leaves and pollen. Among the more highly-expressed Type I genes were *PpeMADS71*, an Mβ expressed primarily in roots (5.7 FPKM), and *PpeMADS39*, an Mα expressed only in fruits (3.6 FPKM). Several other genes showed low-level expression across multiple tissues (e.g. *PpeMADS06, PpeMADS31* and *PpeMADS78*). It should be noted that we did not specifically sample female gametophyte tissue, the location of most Type I gene expression in *Arabidopsis*.

In contrast to the extremely low expression of Type I MADS-box genes (0.4 FPKM averaged over all genes and tissues), expression of Type II genes was markedly higher (8.9 FPKM averaged over all genes and tissues). Only *PpeMADS01* (MIKC*), *PpeMADS04* (*AGL17*) and *PpeMADS62* (*AGL6*) showed no expression in any tissue examined. The greatest number of Type II MADS-box genes was observed in roots (32 genes), followed by young leaves (30), fruit (27), expanded leaves (26), pollen (23), and cotyledon/embryo tissue (17).

We used average linkage clustering to group Type II genes by their tissue-specific expression patterns. A group of genes containing *SEP* and *AG* subfamily members was expressed almost exclusively in fruits, while a group of four *SVP/AGL24*-like genes constituted the most highly-expressed genes in cotyledon + embryo tissue. *FLC*, *SOC1*, *SVP/AGL24* and *AP1/FUL* family members were highly expressed in leaves and roots. Genes with root-only expression included the *AGL17* subfamily members *PpeMADS59* and *PpeMADS47*, as well as the *AGL12* subfamily member *PpeMADS46*. As expected, expression of the MIKC* genes was restricted mainly to pollen, as was expression of *AGL15* and *PI* orthologs. Floral tissue was not represented in our RNA-seq read sets, precluding analysis of ABCDE-type floral homeotic gene expression in peach flowers. Nonetheless, genes from each of the ABCDE gene categories were expressed in multiple peach tissues.

### Gene expression during the short-day transition

**Figure 7:**
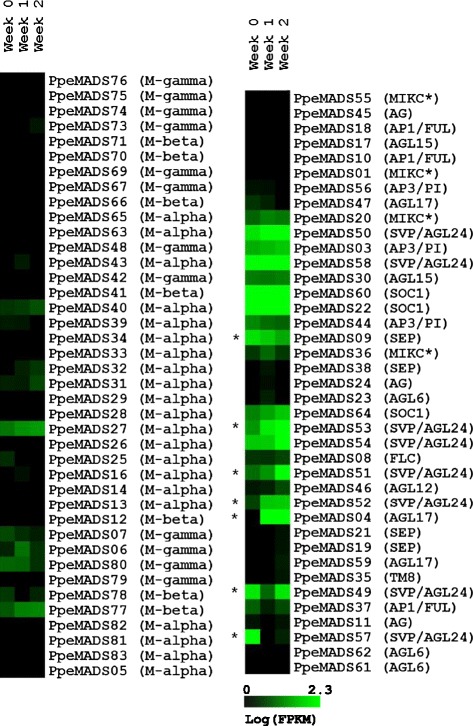
**Expression profiles of Type I (left) and Type II (right) MADS-box genes from peach apical shoots at 0, 1 and 2 weeks after the transition to short days.** FPKM expression values were log-transformed, and genes were grouped by average linkage clustering (see Methods). Asterisks denote genes whose expression level changed significantly over the course of the two-week experiment.

In a second RNA-seq experiment, we quantified MADS-box gene expression in shoot apices before and after the transition to short day dormancy-inducing conditions (Figure 7). Seven Type II genes exhibited significant expression changes in the two weeks following the short-day transition, indicating that these genes may regulate the earliest stages of growth cessation, terminal bud set and endodormancy establishment.

The *SVP* ortholog *PpeMADS57* was strongly down-regulated, as was the *SEP* family member *PpeMADS09. PpeMADS49* (*DAM5*) was down-regulated at week one and returned to its baseline by week two. Three additional *DAM* genes (*PpeMADS51* [*DAM3*], *PpeMADS52* [*DAM6*] and *PpeMADS53* [*DAM2*]) were significantly up-regulated, and a similar trend was observed for *PpeMADS50* (*DAM4*) and *PpeMADS54* (*DAM1*). Among the *DAM* genes, the greatest magnitude of response was observed in *PpeMADS51* (*DAM3*), whose expression increased 45-fold over the two-week interval. Expression of *PpeMADS04* from the *AGL17* subfamily also increased significantly from 0 to 137.15 FPKM during this time. The *FLC* subfamily member *PpeMADS08* was expressed at low levels throughout the experiment and showed no significant change in the two weeks following the short day transition.

## Discussion

### Type I and MIKC genes

We identified 40 Type I MADS-box genes and 39 MIKC MADS-box genes (4 MIKC* and 35 MIKC^c^ ) in peach. The phylogenetic relationships, chromosomal distribution and expression patterns of these two gene families were quite different. Most Type I genes appeared to have arisen through tandem duplications after the divergence of the *Arabidopsis* and peach lineages. They generally formed species-specific clades and clustered in tandem-duplicated groups on individual chromosomes [48,49]. In contrast, most MIKC subfamilies contained members from both species and appear to have been present in the most recent common ancestor of *Arabidopsis* and peach.

Differing patterns of Type I and MIKC gene evolution are not unique to peach and *Arabidopsis* but have recently been documented in MADS-box genes from 24 sequenced plant genomes [49]. Evidence suggests that MIKC genes mainly increase in number following periodic whole genome duplication events [50], whereas Type I genes experience faster rates of birth and death related to tandem duplication and loss [48].

Despite their possession of a similar ~58 amino acid DNA-binding MADS domain, Type I and MIKC MADS-box genes actually share few common features. Type I genes have a very simple gene structure, generally consisting of only a single exon. Yeast two-hybrid screens in *Arabidopsis* suggest that many Type I proteins do not interact with other MADS-box proteins [51]. MIKC genes have a far more complex structure, containing up to 10 exons and three additional domains. Their protein products interact to form multimeric complexes, including the double dimers that specify floral organ identity in *Arabidopsis* [52-54].

The dosage imbalance that results from duplication of only one gene in a multi-protein complex is thought to incur a fitness cost [55]. As a consequence, one member of a gene pair that results from tandem duplication is often removed by purifying selection if its protein product functions as part of a higher level complex [56]. Genes that are less connected are not subject to the same dosage constraints and tend to undergo retention and subfunctionalization following tandem duplication. These trends are borne out in the patterns of evolution exhibited by Type I genes (relatively unconnected) and MIKC genes (highly connected). Exceptions occur, particularly within the *SVP/AGL24* and *FLC* families (see below).

Connectedness may not be the only feature that drives differences in Type I and MIKC phylogenies. Given their short, simple structure, Type I genes may be more likely to be copied intact and in frame during tandem or segmental duplications. It has also been suggested that they exhibit particularly high transposition frequencies, although little direct evidence of transposition exists [49,57]. Their involvement in reproduction, female gametophyte development, and interspecific incompatibility may also promote retention and sub/neofunctionalization [23,49]. Whatever the underlying causes, the partitioning of Type I genes into species-specific clades limits the confidence with which we can functionally annotate peach Type I genes based on sequence similarities with *Arabidopsis* Type I genes.

### Type I gene expression

Type I and MIKC genes generally differ in their tissue-specific expression patterns. In *Arabidopsis*, Type I gene expression is almost invariably low, detectable only with next generation sequencing or RT-PCR rather than blots or arrays [57,58]. *Arabidopsis* Type I genes are primarily expressed in the female gametophyte, developing embryo and early endosperm, whereas MIKC^c^ and MIKC* genes are primarily expressed in the sporophyte and male gametophyte, respectively [49].

We found very low levels of Type I gene expression in the sporophyte and male gametophyte tissues we sampled. In the tissue-specific expression dataset (Figure 6), fourteen Type I genes showed no expression, nineteen were expressed at levels lower than 1 FPKM and only seven were expressed at levels higher than 1 FPKM in any tissue. Similar results were seen in the short day transition dataset (Figure 7), where only nine Type I genes showed expression greater than 1 FPKM in shoot apices. The interpretation of such low FPKM values is problematic. On the one hand, 1 FPKM is a typical threshold used to separate expressed from non-expressed genes in RNAseq experiments [59,60]. On the other hand, transcription factors such as the MADS-box proteins can exert their effects at very low expression levels. Low expression of Type I genes probably has little biological relevance, but given evidence that they may influence flowering time in *Arabidopsis* [61], significant roles for Type I genes in the peach sporophyte cannot be ruled out.

Five Type I genes were expressed at levels higher than 1 FPKM in both RNAseq datasets: *PpeMADS27* and *PpeMADS31* (Mα), *PpeMADS0*6 and *PpeMADS80* (Mγ) and *PpeMADS78* (Mβ). Among these, *PpeMADS27* is perhaps the most interesting, showing expression in all tissues examined and reaching expression levels as high as 19.3 FPKM in young leaves. *PpeMADS27* is most similar to *AGL102*, whose expression has been reported in chalazal endosperm [58] and whose interaction partners include the Mβ genes *AGL78*, *AGL82* and *AGL103* [51]. Members of the *AGL82* lineage (*PpeMADS77* and *PpeMADS78*) were among the more highly expressed Type I peach genes, suggesting that a similar Mα/ Mβ interaction may also occur in peach.

### MIKC* gene expression

In higher plants, MIKC* genes group into the S- and P-clades, members of which form interclade heterodimers and control pollen maturation in *Arabidopsis* [62]. Peach has two members of each clade, *PpeMADS01* and *PpeMADS55* in the S-clade and *PpeMADS20* and *PpeMADS36* in the P-clade. We saw no expression of *PpeMADS01* in any tissue, perhaps indicating that it has become a pseudogene or that it is expressed during a stage of pollen development that we did not sample. The other S-clade gene, *PpeMADS55,* was expressed at high levels (57 FPKM) in pollen and very low levels elsewhere. In two independent RNAseq experiments, P-class genes were highly expressed in pollen (32–76 FPKM) but also expressed in the sporophyte (up to 11.8 FPKM). Expression of MIKC* genes outside the microgametophyte has been documented in non-seed plants [17], but does not appear to occur in *Arabidopsis* and rice [62]. As with persistent low-level expression of Type I genes throughout the plant, the functional significance of low MIKC* expression in the sporophyte remains unclear.

### Floral homeotic genes

MIKC^c^ MADS-box genes from the A-, B-, C-, D- and E-classes function as floral homeotic genes in angiosperms, specifying floral meristem and floral organ identity [53,63,64]. In general, the number and phylogenetic relationships among peach floral homeotic genes are similar to those reported for other eudicot species [18,26,27]. While we did not evaluate homeotic gene expression in flowers, we did measure the expression of A-, B-, C-, D- and E-class genes in other peach tissues.

The *Arabidopsis* A-class gene *AP1*, along with its partially redundant homolog *CAL*, helps to specify floral meristem identity and to direct the development of sepals and petals [65]. It is closely related to a third gene, *AGL79*, whose function is largely unknown [66]. Also in the *AP1* clade is *FUL*, an *Arabidopsis* gene that acts redundantly with *AP1* to determine floral meristem identity and plays additional roles in fruit and leaf development [65]. Peach has two *FUL* orthologs, *PpeMADS37* and *PpeMADS18. PpeMADS37* was expressed at high levels in fruit, roots, and leaves, while *PpeMADS18* showed little expression in any tissue sampled. A third subfamily member, *PpeMADS10,* was highly expressed in roots and leaves.

B-class MADS box genes include *AP3* and *PI*, which form obligate heterodimers in *Arabidopsis* and specify the identity of petals and stamens [65,67]. The clade contains two members in *Arabidopsis* but three in many other eudicots, including peach: *PpeMADS03, PpeMADS56* and *PpeMADS44*. Expression of *PpeMADS56* was restricted almost entirely to pollen, likely indicating the presence of stamen tissue in the pollen sample rather than expression of this gene in the male gametophyte. *PpeMADS03* was expressed throughout the plant, particularly in fruit, pollen and young leaves, and *PpeMADS44* was found in all tissues except the embryo.

*AG* performs the C-class function of stamen and carpel specification in *Arabidopsis* and is a member of a subfamily that also includes the D-class ovule identity genes *SHP1*, *SHP2* and *STK* [65]. The *AG* subfamily has three members in peach: *PpeMADS24*, *PpeMADS11* and *PpeMADS45*, all of which were highly expressed in fruit.

The partially redundant E-class genes *SEP1-4* assist in the formation of higher order complexes among other floral homeotic MADS-box proteins [65]. Their tomato and strawberry orthologs also function in fruit development and ripening [68,69]. Peach has four members of the *SEP1-4* clade (*PpeMADS19*, *PpeMADS38*, *PpeMADS21* and *PpeMADS09*), most of which were highly expressed in fruit and showed little expression elsewhere. It has been reported that genes from the *AGL6* subfamily also exhibit E-class activity. This family contains 2 members (*AGL6* and *AGL13*) in *Arabidopsis* and 3 members (*PpeMADS23*, *PpeMADS61* and *PpeMADS62*) in peach. Expression of these genes was negligible in all tissues we examined.

### Other MIKC^c^ gene families

The *AGL17* clade, containing 4 genes in *Arabidopsis* (*ANR1*, *AGL16*, *AGL17* and *AGL21*), has received significant attention for its role in controlling lateral root growth in response to nutrients [70,71]. These genes are largely root-expressed in *Arabidopsis*, although *AGL16* is also expressed in leaves and stems, where it plays roles in stomatal development and flowering time regulation [13,72,73]. Recently, *AGL17* has also been shown to function downstream of *CONSTANS* in the photoperiodic floral-induction pathway [74]. Peach has three members of this clade: *PpeMADS59*, *PpeMADS47*, and *PpeMADS04,* which was strongly induced under short days (see below). Both *PpeMADS59* and *PpeMADS47* were expressed almost exclusively in roots, while *PpeMADS04* was expressed in apical shoots only following exposure to short days.

*AGL12* constitutes its own subfamily in *Arabidopsis* and is highly expressed in roots, where it influences root meristem proliferation through its effects on auxin and cell cycle regulation [75]. It has been implicated in regulation of the floral transition, and its rice ortholog plays a role in stress response [75,76]. The single peach ortholog, *PpeMADS46*, was expressed almost exclusively in roots. It is interesting to note that roots expressed the largest number of different MADS-box genes, both Type I and MIKC, of all peach tissues examined. While MADS-box genes have received most attention for their role in floral development, they appear to have multiple, less appreciated functions belowground. For example, Moreno-Risueno *et al.* have recently demonstrated that oscillating expression of SOC1, SHP1 and SHP2 is involved in the establishment of *Arabidopsis* lateral root initiation sites [77].

*AGL18* and *AGL15* are expressed in the *Arabidopsis* endosperm and embryo, respectively, and also appear to function in the floral transition [13,65]. *AGL18* is unique in being the only MIKC^c^ gene expressed in an *Arabidopsis* gametophyte: it is found at high levels in pollen [16]. Peach members of this subfamily (*PpeMADS17* and *PpeMADS30*) were also highly expressed in pollen.

*Arabidopsis* has no members of the ancient *TM8* clade, but members are present in most other sequenced eudicots, including tomato, cucumber, poplar, grapevine and peach [64]. While their functions are poorly understood, expression data suggest a role for *TM8* subfamily members in flower development [18,78]. The peach *TM8* ortholog (*PpeMADS35*) showed minimal expression in all tissues examined and is perhaps chiefly expressed in floral tissues, as has been reported for grapevine.

*SOC1* integrates information from multiple flowering time pathways in *Arabidopsis* and, together with *AGL24*, activates the flowering promoter *FT* [20,79]. It is also expressed elsewhere in the plant, particularly in the roots, where it may function in nutrient deficiency response [71]. In addition to *SOC1* itself, the clade contains five more genes in *Arabidopsis*: *AGL14*, *AGL19*, *AGL42*, *AGL71* and *AGL72. AGL14* has been reported mainly in the roots [80], while *AGL19* is induced by cold and promotes flowering in vernalized plants through a non-*FLC* pathway [81]. *AGL42*, *AGL71* and *AGL72* have also been shown to promote flowering through a gibberellin-dependent pathway [82]. Peach has single orthologs of *SOC1* (*PpeMADS22*), *AGL14/19* (*PpeMADS64*), and *AGL42/71/72* (*PpeMADS60*), all which were expressed at high levels in roots and leaves. None of these genes showed significant changes in expression after two weeks under short day conditions.

### FLC and SVP/AGL24 subfamilies

The two remaining MIKC^c^ subfamilies, *SVP/AGL24* and *FLC*, are best known for their roles in *Arabidopsis* flowering time regulation. It is within these last two families that we see the greatest differences in gene number and phylogeny among *Arabidopsis*, peach, and other woody perennials.

The *SVP*/*AGL24* subfamily contains only two members in *Arabidopsis*: the flowering repressor *SVP* and the flowering promoter *AGL24* [26]. Previous reports indicate that this subfamily is expanded in woody perennials [18,27], and our work suggests that multiple expansions have occurred within different branches of the *SVP*/*AGL24* subfamily over the course of plant evolution (Figure 4). While peach contains the six tandem-duplicated *DAM* genes (*PpeMADS49* through *PpeMADS54*) that are most closely related to *AGL24,* poplar and grapevine exhibit expansions within a separate branch of the subfamily that has no *Arabidopsis* members and a single member in peach (*PpeMADS58*). The grouping of these genes in species-specific lineages suggests that the expansions have occurred independently.

Within the main poplar *SVP/AGL24* expansion, three genes (*PtMADS27, 28* and *29*) are tandemly-arranged on chromosome VII, while two genes (*PtMADS4*7 and *48*) are closely adjacent to one another on a syntenic region of chromosome XVII [83]. This pattern suggests a complex history of both tandem and whole-genome duplications. Within the main grapevine *SVP/AGL24* expansion (*VvMADS3*,*4*, and *5*), two genes are located approximately 2 Mb apart on chromosome III, and one is located on chromosome 15 (*Vitis* genome data retrieved from http://www.phytozome.net on Nov. 29, 2014).

It is interesting to note that poplar, grapevine, peach and *Arabidopsis* each contain only one true *SVP* ortholog: no expansions appear to have occurred within the *SVP* subclade itself. *SVP/AGL24* homologs have been referred to as *DAM* genes in several perennial species and are implicated in endodormancy regulation [3,5,84,85]. Nonetheless, given their multiple, independent evolutionary origins, *DAM* genes from different species are unlikely to regulate dormancy in an strictly identical manner.

Expansions have also been reported within the *FLC* subfamily, although not in peach, which contains only a single subfamily member (*PpeMADS08*). The family is expanded in *Arabidopsis* by the presence of the five tandemly-duplicated *MAF* genes [86] and in poplar by a group of five *FLC*-like genes that form a separate subclade in our phylogeny (Figure 5). Four of these genes are tandemly-arranged on poplar chromosome III, while the fifth is found in a syntenic region of chromosome I [83]. Again, independent *FLC* subfamily expansions have occurred in poplar and *Arabidopsis*, and there is no reason to conclude that *FLC*-like genes function identically across species. Indeed, while a poplar *FLC* homolog decreased in expression during the transition to endodormancy [87], our single peach *FLC* homolog showed no expression change after two weeks under dormancy-inducing conditions. It is likely that different perennial species respond to dormancy-inducing conditions using a broadly similar genetic toolkit whose specific genes function in subtly different ways.

The questions remains, why have multiple tandem duplicates arisen and been retained within the *SVP/AGL24* and *FLC* subfamilies in numerous plant lineages? The retention of tandem duplicates does not conform to the typical pattern of fractionation seen in highly-connected MIKC^c^ genes. Do these genes perhaps function as homodimers, freeing them from gene dosage constraints? In a yeast two-hybrid study, *AGL24* formed homodimers, while *SVP* – for which we found no evidence of tandem duplicate retention - did not [51]. In the same study, *FLC* exhibited no interactions with any other MIKC^c^ proteins, and independent evidence suggests that *FLC* functions as part of a multi-protein complex containing at least two copies of the *FLC* protein itself [88]. Perhaps differences in connectivity and interaction among the *SVP*, *AGL24* and *FLC* gene products have permitted retention and sub-/neo-functionalization of duplicates only within the latter two subfamilies.

## Conclusions

Peach contains 79 MADS-box genes distributed across its eight chromosomes, often present in clusters of two or more genes. Most Type I genes appear to have arisen through relatively recent tandem duplications, whereas most Type II genes appear to have increased following whole genome duplication events. An exception to the latter rule occurs in the dormancy-related *FLC* and *SVP/AGL24* Type II subfamilies, in which species-specific tandem duplicates have been retained across a variety of perennial species. As new plant genomes are sequenced and additional expression data become available, we will undoubtedly learn more about the functions and relationships among these dormancy-related genes. Nonetheless, phylogenetic comparisons and expression data presented here suggest that we should proceed with caution when ascribing the specific functions of *Arabidopsis SVP*, *AGL24* and *FLC* to related genes from other species.

### Availability of supporting data

In addition to the supplementary files listed below, fastq sequence files from all RNA-seq experiments are available at the NCBI Sequence Read Archive (http://www.ncbi.nlm.nih.gov/sra): tissue-specific libraries under SRA053230 and short-day transition libraries under SRP046357. Newick files for all phylogenetic trees are available for download at Dryad (http://datadryad.org/; doi:10.5061/dryad.65k7t).

## Additional files

Additional file 1:
**Manually-curated protein sequences of six** ***FLC*** **-like genes from** ***Populus trichocarpa*** **.**
Additional file 2:
**GFF3 file of MADS-box gene locations in the peach genome.**
Additional file 3:
**Excel file of MADS-box gene expression (FPKM) from tissue-specific and short-day transition RNAseq datasets.**
Additional file 4:
**Peach MADS-box gene names and attributes.**

## References

[1] Horvath D (2009). Common mechanisms regulate flowering and dormancy. Plant Sci.

[2] Rodriguez J, Sherman WB, Scorza R, Wisniewski M, Okie WR (1994). Evergreen peach, its inheritance and dormant behavior. J Am Soc Hortic Sci.

[3] Li Z, Reighard GL, Abbott AG, Bielenberg DG (2009). Dormancy-associated MADS genes from the EVG locus of peach [*Prunus persica* (L.) Batsch] have distinct seasonal and photoperiodic expression patterns. J Exp Bot.

[4] Yamane H, Kashiwa Y, Ooka T, Tao R, Yonemori K (2008). Suppression Subtractive Hybridization and Differential Screening Reveals Endodormancy-associated Expression of an SVP / AGL24-type MADS-box Gene in Lateral Vegetative Buds of Japanese Apricot. J Am Soc Hortic Sci.

[5] Ubi BE, Ban Y (2010). Molecular cloning of dormancy-associated MADS-box gene homologs and their characterization during seasonal endodormancy transitional phases of Japanese pear. J Am Soc Hortic Sci.

[6] Mazzitelli L, Hancock RD, Haupt S, Walker PG, Pont SD, McNicol J (2007). Co-ordinated gene expression during phases of dormancy release in raspberry (*Rubus idaeus* L.) buds. J Exp Bot.

[7] Mathiason K, He D, Grimplet J, Venkateswari J, Galbraith DW, Or E (2009). Transcript profiling in *Vitis riparia* during chilling requirement fulfillment reveals coordination of gene expression patterns with optimized bud break. Funct Integr Genomics.

[8] Asante DK, Yakovlev I, Fossdal CG, Holefors A, Opseth L, Olsen JE (2011). Gene expression changes during short day induced terminal bud formation in Norway spruce. Plant Cell Environ.

[9] Jiménez S, Li Z, Reighard GL, Bielenberg DG (2010). Identification of genes associated with growth cessation and bud dormancy entrance using a dormancy-incapable tree mutant. BMC Plant Biol.

[10] Leida C, Terol J, Martí G, Agustí M, Llácer G, Badenes ML (2010). Identification of genes associated with bud dormancy release in *Prunus persica* by suppression subtractive hybridization. Tree Physiol.

[11] Messenguy F, Dubois E (2003). Role of MADS box proteins and their cofactors in combinatorial control of gene expression and cell development. Gene.

[12] De Bodt S, Raes J, Van de Peer Y, Theißen G (2003). And then there were many: MADS goes genomic. Trends Plant Sci.

[13] Alvarez-Buylla ER, Liljegren SJ, Pelaz S, Gold SE, Burgeff C, Ditta GS (2000). MADS-box gene evolution beyond flowers: expression in pollen, endosperm, guard cells, roots and trichomes. Plant J.

[14] Alvarez-Buylla ER, Pelaz S, Liljegren SJ, Gold SE, Burgeff C, Ditta GS (2000). An ancestral MADS-box gene duplication occurred before the divergence of plants and animals. Proc Natl Acad Sci U S A.

[15] Kofuji R, Sumikawa N, Yamasaki M, Kondo K, Ueda K, Ito M (2003). Evolution and divergence of the MADS-box gene family based on genome-wide expression analyses. Mol Biol Evol.

[16] Gramzow L, Theißen G (2010). A hitchhiker’s guide to the MADS world of plants. Genome Biol.

[17] Kwantes M, Liebsch D, Verelst W (2011). How MIKC* MADS-box genes originated and evidence for their conserved function throughout the evolution of vascular plant gametophytes. Mol Biol Evol.

[18] Díaz-Riquelme J, Lijavetzky D, Martínez-Zapater JM, Carmona MJ (2009). Genome-wide analysis of MIKC^C^-type MADS box genes in grapevine. Plant Physiol.

[19] Kaufmann K, Melzer R, Theißen G (2005). MIKC-type MADS-domain proteins: structural modularity, protein interactions and network evolution in land plants. Gene.

[20] Hemming MN, Trevaskis B (2011). Make hay when the sun shines: the role of MADS-box genes in temperature-dependant seasonal flowering responses. Plant Sci.

[21] Amasino RM (2005). Vernalization and flowering time. Curr Opin Biotechnol.

[22] Jiménez S, Reighard GL, Bielenberg DG (2010). Gene expression of DAM5 and DAM6 is suppressed by chilling temperatures and inversely correlated with bud break rate. Plant Mol Biol.

[23] Masiero S, Colombo L, Grini PE, Schnittger A, Kater MM (2011). The emerging importance of Type I MADS box transcription factors for plant reproduction. Plant Cell.

[24] Zobell O, Faigl W, Saedler H, Münster T (2010). MIKC* MADS-box proteins: conserved regulators of the gametophytic generation of land plants. Mol Biol Evol.

[25] Verde I, Abbott AG, Scalabrin S, Jung S, Shu S, Marroni F (2013). The high-quality draft genome of peach (*Prunus persica*) identifies unique patterns of genetic diversity, domestication and genome evolution. Nat Genet.

[26] Parenicova L, De Folter S, Kieffer M, Horner DS, Favalli C, Busscher J (2003). Molecular and phylogenetic analyses of the complete MADS-box transcription factor family in Arabidopsis: New openings to the MADS world. The Plant Cell..

[27] Leseberg CH, Li A, Kang H, Duvall M, Mao L (2006). Genome-wide analysis of the MADS-box gene family in *Populus trichocarpa*. Gene.

[28] Zhao Y, Li X, Chen W, Peng X, Cheng X, Zhu S (2011). Whole-genome survey and characterization of MADS-box gene family in maize and sorghum. Plant Cell Tissue Organ Cult.

[29] Arora R, Agarwal P, Ray S, Singh AK, Singh VP, Tyagi AK (2007). MADS-box gene family in rice: genome-wide identification, organization and expression profiling during reproductive development and stress. BMC Genomics.

[30] Finn RD, Clements J, Eddy SR (2011). HMMER web server: interactive sequence similarity searching. Nucleic Acids Res.

[31] Altschul S, Gish W, Miller W, Myers EW, Lipman DJ (1990). Basic local alignment search tool. J Mol Biol.

[32] Stanke M, Keller O, Gunduz I, Hayes A, Waack S, Morgenstern B (2006). AUGUSTUS: ab initio prediction of alternative transcripts. Nucleic Acids Res.

[33] Korf I (2004). Gene finding in novel genomes. BMC Bioinformatics.

[34] Voorrips RE (2002). MapChart: Software for the graphical presentation of linkage maps and QTLs. J Hered.

[35] Thompson J, Higgins D, Gibson T (1994). CLUSTAL W: improving the sensitivity of progressive multiple sequence alignment through sequence weighting, position-specific gap penalties and weight matrix choice. Nucleic Acids Res.

[36] Guindon S, Dufayard JF, Lefort V, Anisimova M, Hordjik W, Gascuel O (2010). New Algorithms and Methods to Estimate Maximum-Likelihood Phylogenies: Assessing the Performance of PhyML 3.0. Syst Biol..

[37] Katoh K, Standley DM (2013). MAFFT multiple sequence alignment software version 7: improvements in performance and usability. Mol Biol Evol.

[38] Ronquist F, Huelsenbeck JP (2003). MrBayes 3: Bayesian phylogenetic inference under mixed models. Bioinformatics.

[39] Smeds L, Künstner A (2011). ConDeTri - A content dependent read trimmer for Illumina data. PLoS One.

[40] Trapnell C, Roberts A, Goff L, Pertea G, Kim D, Kelley D (2012). Differential gene and transcript expression analysis of RNA-seq experiments with TopHat and Cufflinks. Nat Protoc.

[41] Eisen M, Spellman P, Brown P, Botstein D (1998). Cluster analysis and display of genome-wide expression patterns. Proc Natl Acad Sci U S A.

[42] Meisel L, Fonseca B, Gonzalez S, Baeza-Yates R, Cambiazo V, Campos R (2005). A rapid and efficient method for purifying high quality total RNA from peaches (*Prunus persica*) for functional genomics analyses. Biol Res.

[43] Audic S, Claverie J (1997). The significance of digital gene expression profiles. Genome Res.

[44] Romualdi C, Bortoluzzi S, D’Alessi F, Danieli G (2003). IDEG6: a web tool for detection of differentially expressed genes in multiple tag sampling experiments. Physiol Genomics.

[45] Holub EB (2001). The arms race is ancient history in Arabidopsis, the wildflower. Nat Rev Genet.

[46] Ruelens P, De Maagd R, Proost S, Theißen G, Geuten K, Kaufmann K (2013). FLOWERING LOCUS C in monocots and the tandem origin of angiosperm-specific MADS-box genes. Nat Commun.

[47] Albert V, Soltis DE, Carlson JE, Farmerie WG, Wall PK, Ilut DC (2005). Floral gene resources from basal angiosperms for comparative genomics research. BMC Plant Biol.

[48] Nam J, Kim J, Lee S, An G, Ma H, Nei M (2004). Type I MADS-box genes have experienced faster birth-and-death evolution than type II MADS-box genes in angiosperms. Proc Natl Acad Sci U S A.

[49] Gramzow L, Theißen G (2013). Phylogenomics of MADS-box genes in plants - Two opposing life styles in one gene family. Biology (Basel).

[50] Veron (2007). Evidence of interaction network evolution by whole-genome duplications: a case study in MADS-box proteins. Mol Biol Evol.

[51] De Folter S, Immink RGH, Kieffer M, Par L, Weigel D, Busscher M (2005). Comprehensive interaction map of the Arabidopsis MADS box transcription factors. The Plant Cell..

[52] Egea-Cortines M, Saedler H, Sommer H (1999). Ternary complex formation between the MADS-box proteins SQUAMOSA, DEFICIENS and GLOBOSA is involved in the control of floral architecture in *Antirrhinum majus*. EMBO J.

[53] Theißen G (2001). Development of floral organ identity: stories from the MADS house. Curr Opin Plant Biol.

[54] Eckardt N (2003). MADS monsters: Controlling floral organ identity. Plant Physiol..

[55] Papp B, Pal C, Hurst LD (2013). Dosage sensitivity and the evolution of gene families in yeast. Nature.

[56] Freeling M (2009). Bias in plant gene content following different sorts of duplication: tandem, whole-genome, segmental, or by transposition. Annu Rev Plant Biol.

[57] De Bodt S, Raes J, Florquin K, Rombauts S, Rouzé P, Theissen G (2003). Genomewide structural annotation and evolutionary analysis of the type I MADS-box genes in plants. J Mol Evol.

[58] Bemer M, Heijmans K, Airoldi C, Davies BH, Angenent GC (2010). An atlas of type I MADS-box gene expression during female gametophyte and seed development in Arabidopsis. Plant Physiol.

[59] ModENCODE Consortium (2010). Identification of functional elements and regulatory circuits by Drosophila modENCODE. Science.

[60] Nagaraj N, Wisniewski JR, Geiger T, Cox J, Kircher M, Kelso J (2011). Deep proteome and transcriptome mapping of a human cancer cell line. Molecular Systems Biology..

[61] Yoo SK, Lee JS, Ahn JH (2006). Overexpression of AGAMOUS-LIKE 28 (AGL28) promotes flowering by upregulating expression of floral promoters within the autonomous pathway. Biochem Biophys Res Commun.

[62] Liu Y, Cui S, Wu F, Yan S, Lin X, Du X (2013). Functional conservation of MIKC*-Type MADS box genes in Arabidopsis and rice pollen maturation. Plant Cell.

[63] Immink RGH, Kaufmann K, Angenent GC (2010). The “ABC” of MADS domain protein behaviour and interactions. Semin Cell Dev Biol.

[64] Heijmans K, Morel P, Vandenbussche M (2012). MADS-box genes and floral development: the dark side. J Exp Bot.

[65] Smaczniak C, Immink RGH, Angenent GC, Kaufmann K (2012). Developmental and evolutionary diversity of plant MADS-domain factors: insights from recent studies. Development.

[66] Ferrándiz C, Fourquin C (2014). Role of the FUL-SHP network in the evolution of fruit morphology and function. J Exp Bot..

[67] Becker A, Theißen G (2003). The major clades of MADS-box genes and their role in the development and evolution of flowering plants. Mol Phylogenet Evol.

[68] Seymour GB, Ryder CD, Cevik V, Hammond JP, Popovich A, King GJ (2011). A SEPALLATA gene is involved in the development and ripening of strawberry (*Fragaria x ananassa* Duch.) fruit, a non-climacteric tissue. J Exp Bot.

[69] Vrebalov J, Ruezinsky D, Padmanabhan V, White R, Medrano D, Drake R (2002). A MADS-box gene necessary for fruit ripening at the tomato ripening-inhibitor (rin) locus. Science.

[70] Zhang H, Forde BG (2000). Regulation of Arabidopsis root development by nitrate availability. J Exp Bot.

[71] Gan Y, Filleur S, Rahman A, Gotensparre S, Forde BG (2005). Nutritional regulation of ANR1 and other root-expressed MADS-box genes in Arabidopsis thaliana. Planta.

[72] Kutter C, Schöb H, Stadler M, Meins F, Si-Ammour A (2007). MicroRNA-mediated regulation of stomatal development in Arabidopsis. Plant Cell.

[73] Hu J-Y, Zhou Y, He F, Dong X, Liu L-Y, Coupland G (2014). miR824-Regulated AGAMOUS-LIKE16 Contributes to Flowering Time Repression in Arabidopsis. Plant Cell.

[74] Han P, García-Ponce B, Fonseca-Salazar G, Alvarez-Buylla ER, Yu H (2008). AGAMOUS-LIKE 17, a novel flowering promoter, acts in a FT-independent photoperiod pathway. Plant J.

[75] Tapia-López R, García-Ponce B, Dubrovsky JG, Garay-Arroyo A, Pérez-Ruíz RV, Kim S-H (2008). An AGAMOUS-related MADS-box gene, XAL1 (AGL12), regulates root meristem cell proliferation and flowering transition in Arabidopsis. Plant Physiol.

[76] Lee S, Woo Y-M, Ryu S-I, Shin Y-D, Kim WT, Park KY (2008). Further characterization of a rice AGL12 group MADS-box gene, OsMADS26. Plant Physiol.

[77] Moreno-Risueno MA, Norman JM V, Moreno A, Zhang J, Ahnert SE, Benfey PN (2011). Oscillating gene expression determines competence for periodic Arabidopsis root branching. Science..

[78] Pnueli L, Abu-Abeid M, Zamir D, Nacken W, Schwartz-Sommer Z, Lifschitz E (1991). The MADS box gene family in tomato: temporal expression during floral development, conserved secondary structures and homology with homeotic genes from Antirrhinum and Arabidopsis. Plant J.

[79] Lee J, Lee I (2010). Regulation and function of SOC1, a flowering pathway integrator. J Exp Bot.

[80] Rounsley SD, Ditta GS, Yanofsky MF (1995). Diverse roles for MADS box genes in Arabidopsis development. Plant Cell.

[81] Schönrock N, Bouveret R, Leroy O, Borghi L, Köhler C, Gruissem W (2006). Polycomb-group proteins repress the floral activator AGL19 in the FLC-independent vernalization pathway. Genes Dev.

[82] Dorca-Fornell C, Gregis V, Grandi V, Coupland G, Colombo L, Kater MM (2011). The Arabidopsis SOC1-like genes AGL42, AGL71 and AGL72 promote flowering in the shoot apical and axillary meristems. Plant J.

[83] Genome T, Torr P, Tuskan AGA, Difazio S, Jansson S, Bohlmann J (2006). The genome of black cottonwood, *Populus trichocarpa* (Torr. &Gray). Science.

[84] Horvath DP, Sung S, Kim D, Chao W, Anderson J (2010). Characterization, expression and function of DORMANCY ASSOCIATED MADS-BOX genes from leafy spurge. Plant Mol Biol.

[85] Sasaki R, Yamane H, Ooka T, Jotatsu H, Kitamura Y, Akagi T (2011). Functional and expressional analyses of PmDAM genes associated with endodormancy in Japanese apricot. Plant Physiol.

[86] Ratcliffe OJ, Kumimoto RW, Wong BJ, Riechmann JL (2003). Analysis of the Arabidopsis MADS AFFECTING FLOWERING gene family : MAF2 prevents vernalization by short periods of cold. The Plant Cell.

[87] Chen K-Y (2008). Type II MADS-box genes associated with poplar apical bud development and dormancy.

[88] Helliwell C, Wood CC, Robertson M, James Peacock W, Dennis ES (2006). The Arabidopsis FLC protein interacts directly in vivo with SOC1 and FT chromatin and is part of a high-molecular-weight protein complex. Plant J.

