# Supplementary material for: A genome-wide analysis of MADS-box genes in peach [Prunus persica (L.) Batsch]
Source: BMC Plant Biol. 2015 Feb 7;15:41. doi: 10.1186/s12870-015-0436-2 (PMC4329201; doi:10.1186/s12870-015-0436-2)
Supplement: Additional file 4: — Peach MADS-box gene names and attributes. [file 12870_2015_436_MOESM4_ESM.docx]

| **Supplementary Table 2.**  Peach MADS-box gene names and attributes. | | | | | | | |  |
| --- | --- | --- | --- | --- | --- | --- | --- | --- |
|  |  |  |  |  |  |  |  |  |
| **Gene name** | **Additional name(s)** | **GenBank accession** | **GDR Transcript ID** | **Chromosome** | **Strand** | **CDS start position** | **Protein length** | **Family** |
| PpeMADS01 | -- | -- | ppa023054m | 1 | + | 35768602 | 334 | MIKC* |
| PpeMADS03 | -- | -- | ppa026083m | 1 | - | 32875211 | 221 | MIKC^c^ |
| PpeMADS04 | -- | -- | -- | 6 | + | 19866626 | 213 | MIKC^c^ |
| PpeMADS05 | -- | -- | ppb022733m | 6 | + | 20249114 | 165 | Mα |
| PpeMADS06 | -- | -- | ppa019749m | 6 | + | 25666832 | 249 | Mγ |
| PpeMADS07 | -- | -- | ppa022799m | 6 | + | 25661232 | 243 | Mγ |
| PpeMADS08 | -- | -- | ppa015338m | 3 | - | 21497876 | 219 | MIKC^c^ |
| PpeMADS09 | MADS7 | EF440352 | ppa010548m | 3 | - | 18623346 | 245 | MIKC^c^ |
| PpeMADS10 | -- | -- | ppa010249m | 3 | - | 18608644 | 271 | MIKC^c^ |
| PpeMADS11 | MADS8 | EU072119 | ppa010578m | 3 | + | 13593518 | 245 | MIKC^c^ |
| PpeMADS12 | -- |  | ppa027181m | 3 | + | 10529378 | 389 | Mβ |
| PpeMADS13 | -- | -- | ppa021508m | 3 | + | 1523303 | 223 | Mα |
| PpeMADS14 | -- | -- | ppa024282m | 3 | + | 1703484 | 232 | Mα |
| PpeMADS16 | -- | -- | ppa016038m | 7 | - | 12850967 | 215 | Mα |
| PpeMADS17 | -- | -- | ppa022973m | 7 | + | 9086647 | 243 | MIKC^c^ |
| PpeMADS18 | MADS1 | EU079377 | ppa010723m | 1 | + | 27954861 | 238 | MIKC^c^ |
| PpeMADS19 | -- | -- | ppa1027139m | 1 | + | 27947267 | 249 | MIKC^c^ |
| PpeMADS20 | -- | -- | ppa017562m | 1 | + | 27204283 | 359 | MIKC* |
| PpeMADS21 | MADS5 | EF440351 | ppa010679m | 1 | + | 22775642 | 240 | MIKC^c^ |
| PpeMADS22 | -- | -- | ppa014428m | 2 | - | 17029252 | 215 | MIKC^c^ |
| PpeMADS23 | -- | -- | ppa019932m | 2 | + | 17015682 | 244 | MIKC^c^ |
| PpeMADS24 | MADS4 | AY705972 | ppa010595m | 4 | + | 3457114 | 244 | MIKC^c^ |
| PpeMADS25 | -- | -- | ppa024816m | 4 | + | 3790238 | 187 | Mα |
| PpeMADS26 | -- | -- | ppa022905m | 4 | + | 3787571 | 209 | Mα |
| PpeMADS27 | -- | -- | ppa017542m | 1 | + | 3427794 | 200 | Mα |
| PpeMADS28 | -- | -- | ppa026127m | 8 | + | 15767101 | 218 | Mα |
| PpeMADS29 | -- | -- | ppa017246m | 8 | + | 15769668 | 240 | Mα |
| PpeMADS30 | -- | -- | ppa021434m | 2 | + | 506948 | 254 | MIKC^c^ |
| PpeMADS31 | -- | -- | ppa026571m | 6 | - | 8810286 | 196 | Mα |
| PpeMADS32 | -- | -- | ppa026464m | 6 | - | 8808572 | 172 | Mα |
| PpeMADS33 | -- | -- | ppa022494m | 8 | - | 16071834 | 378 | Mα |
| PpeMADS34 | -- | -- | ppa026089m | 8 | - | 16076977 | 275 | Mα |
| PpeMADS35 | -- | -- | ppa019906m | 5 | - | 13615686 | 201 | MIKC^c^ |
| PpeMADS36 | -- | -- | ppa015857m | 5 | + | 15841234 | 371 | MIKC* |
| PpeMADS37 | -- | FC862657 | ppa010308m | 5 | + | 16634911 | 255 | MIKC^c^ |
| PpeMADS38 | MADS2 | DQ102369 | ppa010391m | 5 | + | 16626466 | 251 | MIKC^c^ |
| PpeMADS39 | -- | -- | ppa021886m | 5 | - | 18298626 | 222 | Mα |
| PpeMADS40 | -- | -- | ppa024464m | 2 | - | 15000190 | 230 | Mα |
| PpeMADS41 | -- | -- | ppa021970m | 2 | - | 14136507 | 366 | Mβ |
| PpeMADS42 | -- | -- | ppa017207m | 2 | + | 13998378 | 159 | Mγ |
| PpeMADS43 | -- | -- | ppa023670m | 2 | + | 3754018 | 198 | Mα |
| PpeMADS44 | -- | -- | ppa010727m | 7 | + | 17290696 | 235 | MIKC^c^ |
| PpeMADS45 | -- | EF602037 | ppa011140m | 1 | - | 44889611 | 223 | MIKC^c^ |
| PpeMADS46 | -- | -- | ppa015966m | 1 | + | 45011005 | 204 | MIKC^c^ |
| PpeMADS47 | -- | -- | ppa014573m | 1 | - | 45784255 | 257 | MIKC^c^ |
| PpeMADS48 | -- | -- | ppa018168m | 1 | + | 46199123 | 229 | Mγ |
| PpeMADS49 | DAM5 | DQ863251 | ppa010822m | 1 | - | 46351455 | 236 | MIKC^c^ |
| PpeMADS50 | DAM4 | DQ863250 | ppa011123m | 1 | - | 46360655 | 237 | MIKC^c^ |
| PpeMADS51 | DAM3 | DQ863256 | ppa010758m | 1 | - | 46370941 | 240 | MIKC^c^ |
| PpeMADS52 | DAM6 | DQ863252 | ppa010714m | 1 | - | 46341893 | 238 | MIKC^c^ |
| PpeMADS53 | DAM2 | DQ863255 | ppb017585m | 1 | - | 46389117 | 240 | MIKC^c^ |
| PpeMADS54 | DAM1 | DQ863253 | ppa018667m | 1 | - | 46398152 | 242 | MIKC^c^ |
| PpeMADS55 | -- | -- | ppa007816m | 1 | + | 41122758 | 331 | MIKC* |
| PpeMADS56 | MADS10 | EU005663 | ppa012927m | 1 | - | 39775605 | 203 | MIKC^c^ |
| PpeMADS57 | -- | -- | ppa011063m | 6 | + | 18701564 | 228 | MIKC^c^ |
| PpeMADS58 | -- | -- | ppa022274m | 8 | - | 7942765 | 244 | MIKC^c^ |
| PpeMADS59 | -- | -- | ppa024736m | 8 | - | 7379852 | 256 | MIKC^c^ |
| PpeMADS60 | -- | -- | ppa019267m | 2 | - | 24722920 | 217 | MIKC^c^ |
| PpeMADS61 | -- | -- | ppa022942m | 2 | + | 24694324 | 239 | MIKC^c^ |
| PpeMADS62 | -- | -- | ppa014844m | 4 | + | 5488532 | 239 | MIKC^c^ |
| PpeMADS63 | -- | -- | ppa023142m | 6 | + | 9264363 | 210 | Mα |
| PpeMADS64 | -- | -- | ppb018415m | 5 | - | 9868979 | 218 | MIKC^c^ |
| PpeMADS65 | -- | -- | ppa024983m | 1 | + | 6082817 | 199 | Mα |
| PpeMADS66 | -- | -- | ppa020857m | 3 | + | 21437946 | 375 | Mβ |
| PpeMADS67 | -- | -- | ppa022382m | ^*^15 (1) | - | 147037 | 405 | Mγ |
| PpeMADS69 | -- | -- | -- | 15 (1) | - | 155039 | 112 | Mγ |
| PpeMADS70 | -- | -- | ppa013383m | 7 | + | 5532224 | 126 | Mβ |
| PpeMADS71 | -- | -- | ppa018410m | 7 | - | 5661539 | 224 | Mβ |
| PpeMADS73 | -- | -- | ppa021813m | 7 | + | 12936118 | 355 | Mγ |
| PpeMADS74 | -- | -- | ppa014932m | 7 | + | 12921446 | 211 | Mγ |
| PpeMADS75 | -- | -- | ppa025369m | 7 | + | 1292889 | 225 | Mγ |
| PpeMADS76 | -- | -- | ppa024303m | 3 | - | 625362 | 146 | Mγ |
| PpeMADS77 | -- | -- | ppa023499m | 7 | + | 5931577 | 207 | Mβ |
| PpeMADS78 | -- | -- | ppa026256m | 7 | + | 5877963 | 233 | Mβ |
| PpeMADS79 | -- | -- | ppa024403m | 15 (1) | - | 196599 | 368 | Mγ |
| PpeMADS80 | -- | -- | ppa014627m | 1 | + | 44547038 | 177 | Mγ |
| PpeMADS81 | -- | -- | ppa019217m | 6 | + | 15952127 | 449 | Mα |
| PpeMADS82 | -- | -- | ppa022437m | 1 | + | 2261021 | 221 | Mα |
| PpeMADS83 | -- | -- | -- | 4 | - | 4312111 | 553 | Mα |
| ^*^ Scaffold 15 has been mapped to chromosome 1, beginning at position 13,139,480 | | | | | | | | |
